# Hybrid expert system for lifestyle recommendations in hypertensive patients

**DOI:** 10.3389/frai.2026.1794925

**Published:** 2026-03-30

**Authors:** Miguel Angel Valles-Coral, Lloy Pinedo, Richard Injante, Jorge Raul Navarro-Cabrera, Karen Luz Quintanilla-Morales, Sarita Saavedra, Jorge Valverde-Iparraguirre, Flor Enith Leveau-Barrera, Nerida Gonzalez-Gonzalez

**Affiliations:** 1 Grupo de Investigación en Inteligencia Artificial, Facultad de Ingeniería de Sistemas e Informática, Universidad Nacional de San Martín, Tarapoto, Peru; 2 Grupo de Investigación Transformación Digital Empresarial, Facultad de Ingeniería y Negocios, Universidad Privada Norbert Wiener, Lima, Peru; 3 Grupo de Investigación en Promoción y prevención de la salud en diferentes etapas de vida del ser humano, Facultad de Medicina Humana, Universidad Nacional de San Martín, Tarapoto, Peru

**Keywords:** clinical decision support, hybrid expert system, hypertension management, lifestyle recommendations, patient stratification, unsupervised learning

## Abstract

**Introduction:**

Hypertension management requires personalized lifestyle interventions, yet clinical decision-making often relies on manual assessment and limited decision-support tools. This study presents a hybrid clinical decision-support system that integrates unsupervised machine learning with rule-based expert reasoning to generate personalized lifestyle recommendations for hypertensive patients.

**Methods:**

A real-world dataset of 615 clinical records obtained from routine healthcare services was analyzed. A preprocessing pipeline including data imputation, normalization, and dimensionality reduction was applied prior to patient stratification. Principal Component Analysis (PCA) preserved the dominant latent structure of the dataset, followed by K-Means clustering to identify patient profiles. The resulting clusters were integrated into a rule-based inference engine structured across six lifestyle intervention domains: physical activity, stress management, nutrition, sleep patterns, therapeutic adherence, and general health behaviors. Recommendations were generated using a dual-weighting strategy that prioritizes individual patient attributes while incorporating cluster-level contextual information. System performance was evaluated through blind expert validation involving cardiologists and clinical nutritionists.

**Results:**

K-Means clustering identified three clinically interpretable patient profiles with a Silhouette coefficient of 0.5608. Agreement between automated recommendations and expert clinical consensus reached 78.3%, with a Cohen’s Kappa coefficient of 0.742, indicating substantial concordance. No statistically significant differences were observed between system outputs and expert judgments (*χ*^2^ = 8.347, *p* = 0.908).

**Discussion:**

The findings demonstrate that combining unsupervised patient stratification with explicit clinical reasoning enables interpretable and scalable decision support for non-pharmacological hypertension management. This approach may be particularly valuable in healthcare environments with limited labeled data and constrained clinical resources.

## 1 Introduction

Chronic non-communicable diseases represent one of the most critical challenges for contemporary healthcare systems, not only due to their clinical impact but also because of the growing pressure they exert on decision-making processes, resource allocation, and long-term patient monitoring (Kadum et al., 2023; Hajat and Stein, 2018). Among these conditions, arterial hypertension remains a leading risk factor for cardiovascular and cerebrovascular events, accounting for a substantial proportion of preventable morbidity and mortality worldwide (Montagna et al., 2023; Carey et al., 2018; Boehme et al., 2017).

By 2025, nearly 20% of the global adult population is expected to live with hypertension, a condition often referred to as the “silent killer” due to its asymptomatic progression and delayed diagnosis (Yan et al., 2022). Beyond its direct health consequences, uncontrolled hypertension generates significant socioeconomic burdens, particularly in low- and middle-income regions, where healthcare systems face structural limitations in continuous monitoring, patient follow-up, and personalized intervention delivery (Gheorghe et al., 2018; Athanasakis, 2017; Goorani et al., 2024).

From an informatics perspective, this scenario highlights a critical limitation of traditional hypertension management models: the lack of scalable, adaptive, and explainable decision-support mechanisms capable of transforming heterogeneous clinical and behavioral data into actionable recommendations (Andersson et al., 2023). Existing digital interventions—such as web platforms, mobile applications, and remote monitoring systems—often rely on continuous user interaction, high connectivity, or fully supervised learning paradigms, which restrict their applicability in resource-constrained settings (Cajita et al., 2021; Lozano-Flores, 2023).

Recent advances in artificial intelligence have enabled the development of intelligent health systems capable of supporting non-pharmacological interventions through data-driven recommendations. However, most proposed solutions focus on supervised prediction or rule-based guidelines in isolation, frequently overlooking patient heterogeneity and the latent structures embedded in real-world clinical data. In particular, the integration of unsupervised learning for patient stratification with knowledge-driven expert reasoning remains underexplored, despite its potential to enhance personalization while preserving interpretability and clinical trust.

In the Peruvian Amazon region of San Martín, hypertension represents a pressing public health concern (Ruiz-Alejos et al., 2022; Hernández-Vásquez et al., 2023). Local epidemiological evidence suggests that approximately half of hypertensive individuals remain undiagnosed, while a significant proportion of diagnosed patients maintain lifestyle patterns that increase cardiovascular risk (Saucedo et al., 2021). Healthcare services in the region are frequently saturated, limiting the feasibility of sustained, individualized counseling based on clinical follow-up alone.

Motivated by this context, this study proposes a hybrid expert system that combines unsupervised clustering techniques with rule-based clinical inference to generate personalized lifestyle recommendations for hypertensive patients. By leveraging Principal Component Analysis and K-Means clustering to identify distinct clinical–behavioral profiles, and integrating these profiles into an explainable inference engine, the proposed system aims to support scalable, non-pharmacological hypertension management without requiring extensive labeled datasets or continuous digital engagement.

The main contributions of this work are threefold: (i) the development of an unsupervised patient stratification framework grounded in real clinical and behavioral data; (ii) the design of a hybrid inference architecture that balances individual-level information with group-level patterns; and (iii) the empirical validation of automated recommendations against expert clinical judgment. Together, these contributions position the proposed system as a practical decision-support tool for hypertension management in technology-limited healthcare environments (Peacock et al., 2022; Schoenthaler et al., 2017; Choudhry et al., 2022).

Based on the limitations identified in existing digital health solutions for hypertension—particularly the reliance on supervised learning, continuous user engagement, or application-centric designs—there is a clear need for decision-support systems that can operate with heterogeneous clinical data, limited labeling and explicit reasoning mechanisms. Addressing this gap, the present study introduces a hybrid expert system that integrates unsupervised patient stratification with rule-based clinical inference to generate personalized lifestyle recommendations. The proposed approach is designed to balance scalability, interpretability, and clinical validity, making it suitable for deployment in resource-constrained healthcare environments.

The remainder of this paper is organized as follows. Section 2 reviews related work on digital and AI-based hypertension management systems. Section 3 describes the materials and methods, including data collection, preprocessing, clustering methodology, and expert system design. Section 4 presents the experimental results and validation outcomes. Section 5 discusses methodological implications, clinical relevance, and limitations. Finally, Section 6 concludes the study and outlines future research directions.

## 2 Related work

Digital health interventions for hypertension management have evolved along three dominant lines: (i) mobile and web-based clinical support applications, (ii) data-driven machine learning systems for risk identification and personalization, and (iii) conversational or IoT-enabled platforms that facilitate self-monitoring and behavior change. Although these approaches have advanced non-pharmacological care, they often differ in scalability requirements, interpretability, and dependence on labeled data, leaving open a practical gap for hybrid and explainable decision-support systems in resource-limited settings.

### 2.1 Mobile and guideline-driven applications

Several studies have focused on mobile applications designed to operationalize clinical recommendations through structured interfaces. In Cuba, Olivera Solís et al. (2022) developed an Android application for the diagnosis, evaluation, and treatment of hypertension, integrating clinical calculators and both pharmacological and non-pharmacological guidance. The system achieved high usability among specialists, suggesting that guideline-based digitization can effectively support primary care workflows. Similarly, other mobile proposals emphasize user-facing tools that improve access to recommendations but typically assume sustained interaction, adequate digital literacy, and stable device availability—constraints that can limit adoption in low-resource regions.

A more specialized application direction targets dietary recommendation, as in the DASH diet recommender implemented as an Android app in Sookrah et al. (2019). By combining content-based filtering with an artificial neural network, this system personalized dietary plans using demographic, clinical, and lifestyle variables and reported strong performance in food classification and perceived user benefit. While these results are promising, this line of work is commonly shaped around domain-specific recommendation (e.g., diet) and frequently relies on either high-performing supervised components or continuous user engagement to maintain personalized guidance.

### 2.2 Machine learning systems for diagnosis and variable relevance

A second line of research employs supervised machine learning to predict hypertension status or identify influential determinants, occasionally extending outputs into lifestyle suggestions. Islam and Shamsuddin (2021) proposed a CNN-based diagnostic model that achieved moderate predictive performance and highlighted variables such as age, BMI, smoking, education, occupation, and diabetes as relevant features, translating these findings into lifestyle-oriented guidance. This approach demonstrates how supervised models can uncover predictors and support decision-making. However, it also illustrates two recurrent limitations in practice: (i) performance is often constrained by data size and labeling quality, and (ii) model outputs may be less transparent to clinicians unless complemented by explainability or rule-based reasoning.

In many clinical contexts—particularly in routine hospital services—fully supervised pipelines are difficult to sustain due to incomplete records, heterogeneity in measurement protocols, and limited availability of reliably labeled datasets at scale. Consequently, alternative strategies that can exploit unlabeled or partially labeled data while preserving interpretability remain valuable for deployment-oriented health informatics.

### 2.3 Conversational agents and digital therapeutics for behavior change

A third approach emphasizes behavioral intervention delivery through conversational interfaces or structured digital therapies. In Japan, a randomized study evaluated a non-pharmacological digital therapy delivered via a mobile app with chatbot components, collecting lifestyle and blood pressure information and generating personalized recommendations through cloud processing (Kario et al., 2021; Colakoglu et al., 2025). This model aligns with contemporary “digital therapeutics” trends by integrating education, behavior change modules, and self-assessment, with strong acceptance and measurable improvements in blood pressure control.

Similarly, the WhatsApp-based chatbot evaluated in South Africa (Mash et al., 2022) showed that conversational systems can reach large populations, enhance self-management behaviors, and provide a cost-effective complement to conventional care—particularly during access disruptions such as the COVID-19 pandemic. These studies collectively demonstrate the feasibility of scalable intervention delivery. Nonetheless, they may depend on connectivity, continuous participation, and sustained engagement, and they do not necessarily address the informatics challenge of structuring heterogeneous clinical variables into explicit, auditable reasoning aligned with expert clinical criteria.

### 2.4 IoT-enabled monitoring and hybrid sensing–classification models

Hypertension prevention and detection have also been explored using IoT-based monitoring combined with machine learning classification. In Mexico, Alor-Hernandez et al. (2022) integrated wearable sensing with a J48-based classification approach using Google Fit™ data streams, enabling early detection and individualized monitoring. IoT-enabled solutions expand the data ecosystem and can improve continuity of care, yet they typically require device availability, integration infrastructure, and sustained connectivity—conditions that may not be met in peripheral healthcare environments.

### 2.5 Gap and positioning of this work: unsupervised stratification + rule-based reasoning

Across the reviewed literature, international studies increasingly combine AI and digital platforms to support non-pharmacological hypertension management; however, most solutions adopt either user-facing applications (apps/chatbots) or supervised models for prediction and personalization. In contrast, fewer approaches explicitly integrate unsupervised learning for patient stratification with a rule-based expert system capable of producing explainable recommendations grounded in clinical thresholds and behavioral criteria. This integration is particularly relevant in real-world settings where labeled datasets are limited, patient profiles are heterogeneous, and clinicians require transparent logic to trust automated outputs. Recent advances in explainable clinical decision-support systems and hybrid AI architectures for chronic disease management further support the translational relevance of interpretable and resource-aware digital health solutions.

Moreover, in Latin America—and especially in Peru—the available scientific evidence is comparatively scarce and frequently concentrated on grey literature or on isolated AI tasks (e.g., diagnosis/prediction) rather than full decision-support pipelines that combine profiling, inference, and expert validation. The present work addresses this gap by proposing a hybrid architecture that: (i) identifies clinically meaningful profiles using unsupervised clustering, (ii) encodes expert knowledge into an auditable inference engine to generate lifestyle recommendations, and (iii) validates system outputs against interprofessional expert consensus, supporting deployment in resource-limited healthcare settings such as the San Martín region.

## 3 Materials and methods

This section describes the study design, data collection procedures, preprocessing strategy, clustering methodology, expert system architecture, and validation framework adopted to develop and evaluate the proposed hybrid decision-support model.

This quasi-experimental, longitudinal descriptive study developed an expert system to provide personalized healthy lifestyle recommendations for hypertensive patients treated in the nutrition service of the San Martín region. Classified as applied research, it integrates data analysis and machine learning algorithms on structured clinical records and behavioral patterns to optimize evidence-based non-pharmacological interventions.

The study population comprised patients diagnosed with arterial hypertension treated in the nutrition service of Hospital II-2 Tarapoto (San Martín region), with a reference monthly population of 3,246. Based on finite population sampling (95% confidence, *p* = 0.5, 4% margin of error), the minimum required sample was 384, expanded to 623 records to improve representativeness and segmentation robustness. Inclusion criteria were a confirmed diagnosis of arterial hypertension, willingness to complete follow-up, and signed informed consent. Exclusion criteria included secondary hypertension, decompensated chronic diseases, cognitive impairment, or refusal to participate, ensuring a representative sample for the study objectives.

### 3.1 Data collection instrument

A structured questionnaire with 45 variables was designed in two sections. The first gathered sociodemographic (sex, age, education, occupation), anthropometric (weight, height), lifestyle (smoking, alcohol, sleep, screen time, physical activity), dietary preferences/restrictions, and functional capacity self-assessment. The second recorded clinical and biochemical data, including hypertension history, comorbidities (diabetes, dyslipidemia), medication use, and lab values (glucose, cholesterol, triglycerides, hemoglobin, hematocrit).

The instrument, developed with clinical nutrition and public health experts, was validated by expert judgment (CVI = 0.91) and factor analysis (KMO = 0.82, Bartlett *p* < 0.001), identifying five factors explaining 67.4% of variance. Internal consistency was high (Cronbach’s *α* = 0.85), confirming validity and reliability for similar clinical contexts.

### 3.2 Proposed model

This study proposes a hybrid clinical decision-support model designed to automate the generation of personalized lifestyle recommendations for hypertensive patients by integrating unsupervised machine learning with rule-based expert reasoning. The model was conceived from an informatics perspective, emphasizing modularity, interpretability, and scalability under real-world clinical constraints such as heterogeneous data, partial incompleteness, and limited availability of labeled outcomes.

**Figure 1:**
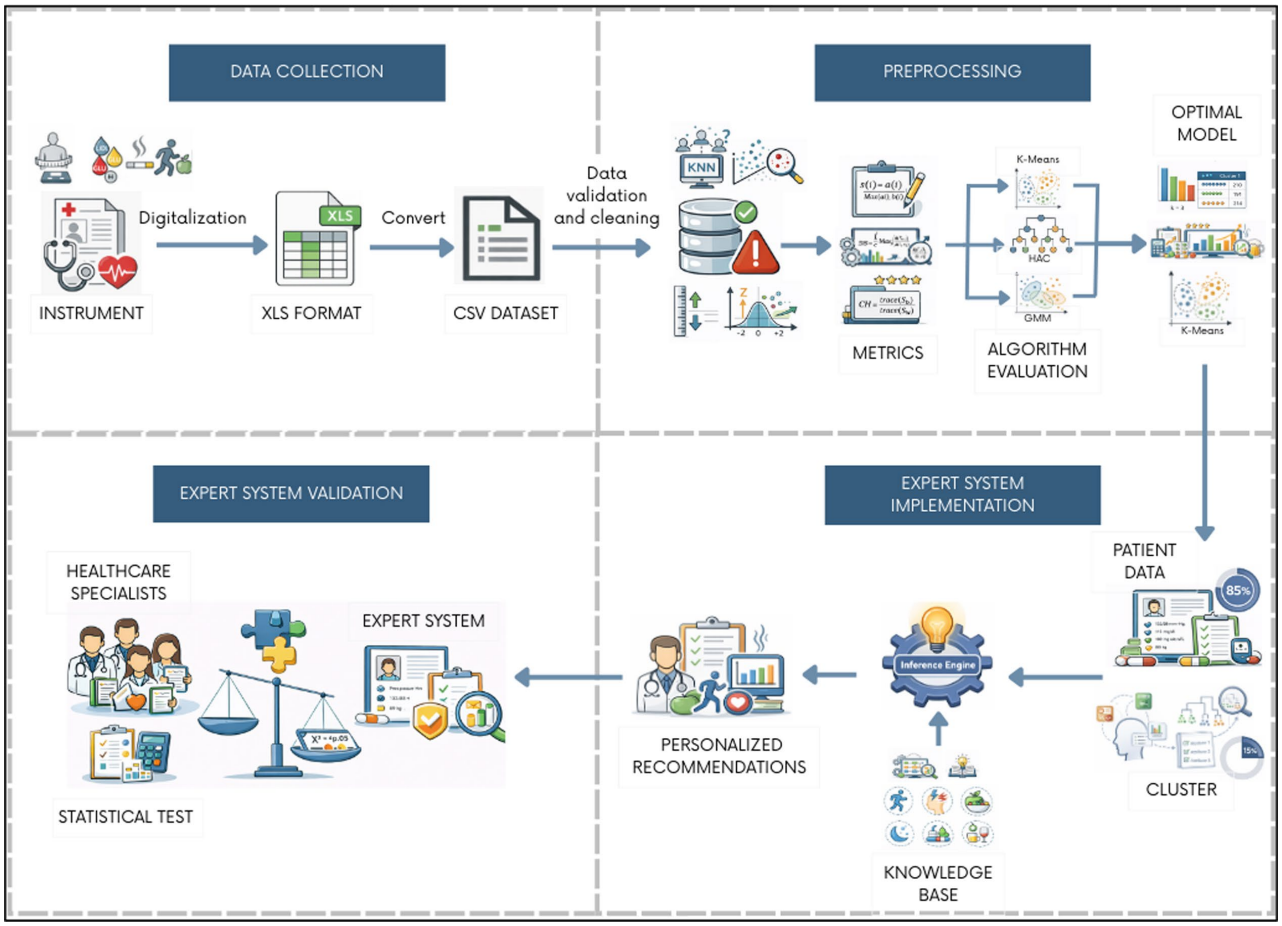
Overall architecture of the proposed model.

As illustrated in Figure 1, the proposed architecture is organized into four interconnected components: (i) data collection, (ii) preprocesing, (iii) expert system implementation, and (iv) expert system validation. Each component operates independently yet exchanges structured outputs with adjacent modules, enabling transparent data flow and facilitating system extensibility.

The data acquisition module ingests structured clinical, anthropometric, biochemical, and behavioral records collected during routine nutrition service consultations. These heterogeneous inputs constitute the raw feature space used for subsequent computational analysis.

The preprocessing and transformation module performs data cleaning, imputation, encoding, and normalization to ensure statistical consistency and algorithmic compatibility. Dimensionality reduction is then applied to mitigate the curse of dimensionality introduced by categorical encoding and to preserve the most informative latent structure of the dataset prior to clustering.

The unsupervised learning module implements patient stratification using Principal Component Analysis (PCA) followed by K-Means clustering. This stage identifies latent clinical–behavioral profiles without relying on predefined labels, allowing the system to exploit real-world data distributions and accommodate inter-patient heterogeneity. The resulting cluster assignments and centroid characteristics serve as group-level descriptors that inform downstream inference.

The expert system module constitutes the core decision-support component. It applies a rule-based inference engine structured across six intervention domains—physical activity, stress management, nutrition, sleep patterns, therapeutic adherence, and general health behaviors. Inference rules are parameterized using clinically validated thresholds and behavioral criteria and are applied through a hybrid weighting strategy that combines individual patient attributes (*α*) with cluster-derived profile characteristics (*β*). This mechanism ensures that recommendations remain personalized while preserving contextual coherence within identified patient groups.

Finally, the validation and evaluation module assesses the agreement between automated recommendations and expert clinical judgment. Outputs generated by the inference engine are independently reviewed by experienced cardiologists and clinical nutritionists, enabling quantitative evaluation through inter-rater agreement metrics and statistical independence testing. This module closes the decision-support loop by anchoring computational outputs to real-world clinical practice.

Overall, the proposed model operationalizes a hybrid artificial intelligence paradigm that bridges data-driven pattern discovery and explicit clinical reasoning. By decoupling patient stratification from recommendation inference and avoiding reliance on extensive labeled datasets, the system offers a practical and explainable solution for non-pharmacological hypertension management in resource-limited healthcare environments.

#### 3.2.1 Phase 1: data collection

After applying the structured instrument, we digitized them in XLS format, converted to CSV, and organized into a database of 623 cases with 52 variables spanning demographic, anthropometric, biochemical, clinical, and behavioral dimensions. Key variables included fasting glucose, lipid profile, hemoglobin, hematocrit, hypertension status, glycemic control, comorbidities, lifestyle habits (alcohol, smoking, physical activity, diet), and antihypertensive treatment indicators (blood pressure values, prescribed regimen, adherence). Data collection followed strict ethical standards with coded identifiers and signed informed consent for scientific use and confidentiality protection.

#### 3.2.2 Phase 2: preprocessing

Before modeling, the dataset was assessed for completeness, consistency, and structural integrity. Of the 8,811 missing values identified (approximately 30% of the dataset), records presenting more than 50% missing information were removed (*n* = 8), resulting in a final working dataset of 615 observations. Variables were classified as numerical or categorical to guide preprocessing strategies.

Missing numerical values were imputed using the K-Nearest Neighbors (KNN) algorithm to preserve multivariate relationships among clinical variables, while categorical variables were imputed using mode substitution. In the case of gender, the modal category in the dataset was female; therefore, the small number of missing entries (*n* = 4; 0.6% of the dataset) were assigned accordingly. Given the minimal proportion of imputed gender values (0.6%), the likelihood of structural bias in clustering or downstream recommendation generation is minimal.

In multiple-choice checklist variables (e.g., food preferences, avoided foods, dietary regimens), blank entries correspond to unmarked options rather than unknown responses, since the instrument records only selected behaviors. Therefore, missing values were mapped to zero to preserve the semantic consistency of multi-hot encoding and avoid artificial category inflation. Additional checks confirmed that this representation did not materially influence cluster formation or behavioral pattern identification.

KNN imputation was selected because the dataset includes heterogeneous and interdependent cardiometabolic variables, where mean or median substitution could distort covariance structure and weaken distance-based pattern detection. The value of k was determined empirically using the square-root heuristic and stability checks across alternative configurations, selecting the setting that preserved cluster coherence while minimizing reconstruction bias. A sensitivity comparison with median imputation confirmed that the overall cluster structure and downstream recommendation patterns remained stable.

Outlier thresholds were defined using clinically plausible ranges and unit consistency checks rather than purely statistical cutoffs. These thresholds were applied to age, anthropometric measurements, biochemical markers, and behavioral risk variables. A systematic coding inconsistency was detected in height values, where a subset of records had been recorded in meters instead of centimeters (*n* = 314); these values were corrected to ensure unit consistency. The preprocessing strategy prioritized correction over deletion, and no additional cases were removed due to physiological value dispersion beyond the previously excluded records with excessive missingness (*n* = 8; 1.3% of the dataset). Numerical variables were standardized using Z-score normalization to prevent scale dominance during distance-based clustering. Pearson correlation matrices were computed to identify significant associations among numerical variables and to support dimensionality reduction decisions prior to clustering.

To address the high dimensionality introduced by categorical encoding and to preserve the intrinsic data structure, Principal Component Analysis (PCA) was applied to 15 key numerical variables, including age, weight, height, systolic and diastolic blood pressure, sleep duration, physical activity frequency, smoking, screen time, hemoglobin, glucose, cholesterol, triglycerides, creatinine, and estimated glomerular filtration rate (eGFR). A variance retention threshold of 90% was adopted to preserve relevant information while reducing noise and redundancy.

PCA was selected due to its capacity to maximize explained variance while maintaining global data structure in a linear and interpretable manner. Given that the dataset consisted primarily of standardized numerical cardiometabolic variables exhibiting moderate linear correlations, PCA was considered suitable for dimensionality reduction prior to distance-based clustering. Unlike nonlinear embedding techniques such as t-SNE or UMAP, which prioritize local neighborhood preservation and visualization, PCA provides reproducible transformations that preserve global variance structure, thereby facilitating stable integration with K-Means clustering and supporting clinical interpretability.

This threshold resulted in the retention of nine principal components, reflecting the heterogeneous multidimensional structure of cardiometabolic and behavioral features within the cohort. The leading components captured clinically interpretable dimensions associated with cardiometabolic risk, behavioral patterns, and hematological markers. A scree plot and corresponding component loadings are provided in the Supplementary material to enhance transparency and reproducibility.

Although the original dataset included both numerical and categorical variables, clustering was performed primarily on PCA-transformed standardized numerical features. Categorical variables were encoded and incorporated within the subsequent rule-based inference layer rather than directly influencing cluster assignment. While mixed-type clustering approaches (e.g., k-prototypes or Gower-distance-based methods) were considered, the predominance of continuous clinical measurements and the objective of modeling latent cardiometabolic structure supported the use of PCA-derived features in conjunction with K-Means.

Three clustering algorithms—K-Means, Hierarchical Agglomerative Clustering (HAC), and Gaussian Mixture Models (GMM)—were evaluated using cluster configurations ranging from k = 3 to k = 6. The optimal clustering solution was selected based on three complementary internal validation metrics.

The Silhouette Index (Equation 1) was used to assess intra-cluster cohesion and inter-cluster separation (Rousseeuw, 1987).


$$s(i)=\frac{\left(b(i)-a(i)\right)}{\max\{a(i),b(i)\}}$$ (1)

The Davies–Bouldin Index (Equation 2) penalized solutions with high intra-cluster dispersion and low inter-cluster separation (Davies and Bouldin, 1979).


$$\mathrm{DB}=\frac{1}{c}\sum_{i=1}^{c}\max_{i\neq j}\{\frac{d(X_{i})+d(X_{j})}{d(c_{i},c_{j})}\}$$ (2)

The Calinski–Harabasz Index (Equation 3) evaluated the ratio between inter-group and intra-group variance (Caliñski and Harabasz, 1974).


$$\mathrm{CH}=\frac{\text{trace}(S_{B})}{\text{trace}(S_{w})}\cdot \frac{n_{p}-1}{n_{p}-k}$$ (3)

Metrics were jointly analyzed to identify the configuration that maximized intra-cluster cohesion, enhanced inter-cluster separation, and minimized overlap between groups. Based on this combined evaluation, K-Means clustering with k = 3 was selected as the optimal stratification model. The resulting clusters were subsequently characterized using descriptive statistics for quantitative variables and frequency distributions for qualitative variables. A variable importance analysis was then conducted to identify the most discriminative features, supporting the clinical and nutritional interpretation of the identified patient profiles.

To assess robustness, the clustering procedure was repeated under multiple random seed initializations and alternative k configurations, confirming stable separation of the dominant profiles and consistent centroid structure across runs.

#### 3.2.3 Phase 3: expert system implementation

The expert recommendation system was implemented as a rule-based clinical decision-support module, designed to translate clustered clinical–behavioral patterns and individual patient attributes into personalized lifestyle recommendations. This phase operationalizes the transition from unsupervised pattern discovery to explicit, interpretable decision logic, ensuring alignment with clinical reasoning while maintaining computational transparency.

The system’s knowledge base was structured across six intervention dimensions: physical activity, stress management, nutrition, sleep patterns, therapeutic adherence, and general health behaviors. For each dimension, discrete intervention levels were defined based on established clinical thresholds, empirical evidence, and expert consensus. These intervention levels constitute the system’s output space and enable standardized yet flexible recommendation generation.

Decision rules were formulated to map combinations of clinical indicators and behavioral variables to specific intervention levels. For example, physical activity recommendations (initiate, maintain, or increase) were derived from the interaction between reported exercise frequency, functional capacity, age, and cardiovascular status. Stress management rules evaluated blood pressure control in conjunction with self-reported stress exposure to determine whether recommendations should aim to maintain current behaviors, reduce stressors, or trigger urgent intervention. Nutritional recommendations were generated based on metabolic indicators—including glucose levels and lipid profile—combined with dietary habits and body mass index.

Sleep-related rules assessed nightly rest duration and its association with blood pressure patterns, classifying recommendations as general or urgent. Therapeutic adherence was inferred from medication compliance indicators and blood pressure control status, producing recommendations categorized as general, maintain, or urgent. Finally, general health behaviors—such as smoking, alcohol consumption, and screen time—were evaluated through frequency-based thresholds and cardiovascular risk criteria to determine whether reduction or urgent intervention was required.

To balance individual personalization with group-level contextualization, the inference engine implemented a dual-weighting strategy. Individual patient features were assigned a weight of *α* = 0.85, while cluster-derived profile attributes contributed a complementary weight of *β* = 0.15. These weights were empirically determined through iterative testing and expert validation, prioritizing patient-specific data while preserving the influence of latent group characteristics identified during clustering. This mechanism prevents overfitting to individual variability and enhances recommendation consistency within clinically similar profiles. To evaluate robustness, alternative weighting configurations were systematically explored during model development, including increased cluster influence (e.g., *β* ≥ 0.25) and reduced cluster influence (*β* ≤ 0.10). Across these configurations, recommendation patterns remained stable, with only minor variations in marginal intervention categories and no structural shifts in high-priority domains. These findings support the robustness of the selected weighting scheme.

From an architectural standpoint, the expert system integrates five functional modules. (i) Preprocessing, which supplies standardized and encoded inputs. (ii) Clustering, which provides patient profile labels and centroid characteristics. (iii) Individual feature extraction, which isolates patient-level indicators. (iv) Group attribute extraction, which retrieves cluster-level descriptors. (v) Logical inference, which applies the rule base and weighting scheme to generate final recommendations. The modular design ensures traceability of decisions, facilitates future rule updates, and supports system extensibility without retraining data-driven components.

#### 3.2.4 Phase 4: expert system validation

The validation phase was designed to assess the agreement between automated recommendations generated by the expert system and the clinical judgment of healthcare professionals experienced in hypertension management. This evaluation aimed to verify reliability as a decision-support tool rather than a diagnostic substitute.

A total of 120 patient cases were selected using stratified sampling proportional to the distribution of the three identified clusters, ensuring heterogeneous representation of clinical–behavioral profiles. For each case, the expert system generated personalized recommendations across the six intervention dimensions.

Five healthcare specialists—two cardiologists and three clinical nutritionists, each with more than 5 years of experience—independently reviewed all selected cases. Evaluations were conducted in a blinded manner; experts had no access to system outputs, cluster assignments, or inferred profiles. Clinical consensus was determined by simple majority (at least three of five specialists).

Agreement between system outputs and clinical consensus was evaluated using Cohen’s Kappa coefficient to quantify concordance strength. Pearson’s chi-square test (*α* = 0.05) was additionally applied to assess statistical independence between automated and expert-generated recommendations.

## 4 Results

This section presents the descriptive characteristics of the study population, the outcomes of the clustering analysis, the structure and distribution of generated recommendations, and the validation results comparing automated outputs with expert clinical judgment.

### 4.1 Descriptive statistics

Table 1 summarizes the cohort’s main sociodemographic, clinical, behavioral, and biochemical characteristics, including the proportion of patients with confirmed hypertension and their distribution by active pharmacological treatment. It also presents means and ranges for key variables such as systolic/diastolic blood pressure, weekly physical activity, and relevant biomarkers, providing a comprehensive profile of the studied population.

**Table 1:** Sociodemographic, clinical, behavioral, and biochemical characteristics of the study cohort.

Variable	Value
Sample size (*n*)	623.00
Confirmed hypertension (%)	84.60
Under pharmacological treatment (%)	80.30
Behavioral and cardiovascular indicators (%)	40.00
Mean systolic BP (mmHg)	142.70
Mean diastolic BP (mmHg)	88.59
BP control target (<130/80 mmHg)	Not achieved
Mean physical activity (days/week)	2.52
Physical activity range (days/week)	1–7

The final dataset included 623 records with 52 variables covering sociodemographic, clinical, anthropometric, biochemical, and behavioral data. Profiles showed marked heterogeneity in age, education, and socioeconomic status, adding robustness to later unsupervised analysis. Of the total, 527 patients (84.6%) had confirmed hypertension, and 423 (80.3% of hypertensive cases) were on pharmacological treatment, while 19.7% were not, suggesting differences in access or adherence and reinforcing the need for lifestyle interventions.

About 40% of variables were behavioral or cardiovascular indicators, enabling assessment of modifiable factors in non-pharmacological hypertension management. Additional biomarkers (hemoglobin, hematocrit, creatinine, eGFR) enriched clinical profiles. Mean systolic/diastolic pressures were 142.70/88.59 mmHg, above international targets (<130/80 mmHg), and mean physical activity was 2.52 days/week (range: 1–7), indicating a general sedentary trend.

Overall, these descriptive results show a clinically heterogeneous hypertensive population with blood pressure values above recommended targets and a high prevalence of modifiable lifestyle-related risk factors. This profile supports the need for structured and personalized non-pharmacological decision support.

### 4.2 Data cleaning and transformation

The data quality assessment found 8,811 missing cells (30% of the dataset). Records with over 50% missing data were excluded, removing eight cases (1.3%), an acceptable trade-off given their systematic absence patterns. Outlier detection revealed inconsistencies in anthropometrics, particularly height, where 314 entries in meters were converted to centimeters; combined with Z-score standardization, this mitigated outlier effects without further removals. Missing numerical values (*n* = 416) were imputed with K-Nearest Neighbors (KNN) to preserve multivariate relationships, and categorical values with mode imputation. Table 2 summarizes missing categorical variables and imputation criteria. After one-hot encoding categorical variables, the final dataset comprised 615 records and 196 columns, justifying dimensionality reduction in subsequent analysis.

**Table 2:** Summary of categorical variables with missing values and applied imputation criteria.

Variable	Missing values	Imputed value
Gender	4	Female
Educational level	13	2
Alcohol consumption	16	Does not drink
Employment status	12	1
Antihypertensive medication	204	1
Glycemic status	39	None
Comorbidities	366	Prostatitis

### 4.3 Cluster analysis via dimensionality reduction

**Figure 2:**
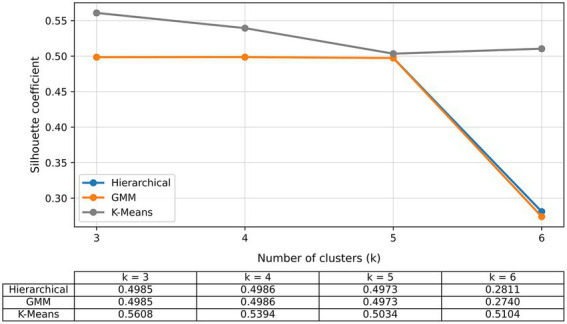
Evolution of the silhouette coefficient across different cluster configurations (*k* = 3 to 6), highlighting the optimal performance achieved by K-means with *k* = 3.

Principal Component Analysis (PCA) substantially reduced dataset complexity while preserving clinically relevant variance. The first component alone explained 84.40% of total variance, suggesting a dominant latent structure driven by co-occurring cardiovascular risk factors. This reduction improved computational efficiency and detection of clinically meaningful patterns. For clustering, three algorithms—K-Means, Agglomerative Hierarchical Clustering, and Gaussian Mixture Models—were tested with k = 3 to 10. K-Means with k = 3 performed best, achieving a Silhouette coefficient of 0.5608, outperforming alternatives whose maximum was 0.4986 with k = 4. The Calinski–Harabasz index also peaked for this configuration, confirming statistical robustness and clinical plausibility (Figure 2).

Beyond statistical superiority, the k = 3 solution provided clinically coherent and interpretable phenotypes that aligned with meaningful cardiovascular–metabolic patterns. Higher k configurations generated fragmented subgroups without clear clinical differentiation, reducing their practical applicability for decision-support purposes. Therefore, the selection of three clusters balanced statistical validity with clinical interpretability.

Descriptive analysis identified three clinically distinct clusters. Cluster 0 (n = 352; 56.5%) comprised patients of intermediate age (51.19 years), moderately elevated weight (70.31 kg), elevated fasting glucose (110.24 mg/dL), acceptable physical activity (2.64 days/week), slightly lower-than-average BP (142.06/88.65 mmHg), elevated hemoglobin (14.08 g/dL), preserved eGFR (81.31 mL/min/1.73m^2^), lower cholesterol (191.72 mg/dL), and slightly elevated triglycerides (130.88 mg/dL). Cluster 1 (n = 264; 42.4%) included older patients (55.90 years), lower weight (65.83 kg), normal glucose (93.40 mg/dL), elevated cholesterol (214.85 mg/dL), reduced physical activity (2.35 days/week), lower hemoglobin (12.67 g/dL), mildly impaired eGFR (77.39 mL/min/1.73m^2^), shorter sleep, and lower smoking prevalence. Cluster 2 (n = 7; 1.1%) was atypical, with greater height (181 cm), weight (79.29 kg), elevated glucose (111.03 mg/dL), high hemoglobin (14.70 g/dL), prolonged sleep, and slightly higher diastolic pressure (89.43 mmHg) with average systolic values.

Although Cluster 2 represented a small proportion of the cohort (*n* = 7; 1.1%), additional stability checks confirmed that these observations consistently grouped together across multiple random initializations and alternative clustering runs. Cluster-wise cohesion metrics indicated that this subgroup exhibited internal similarity distinct from the dominant clusters, suggesting that it does not merely reflect isolated outliers. Nevertheless, given its limited size, findings related to this profile should be interpreted cautiously, and broader generalization would require validation in larger or multi-center datasets.

**Figure 3:**
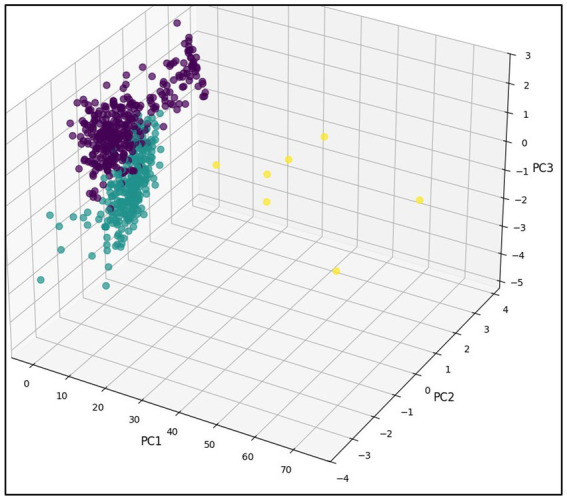
Three-dimensional projection of patient clusters generated via PCA, illustrating separation and distribution across the first three principal components.

Group separation was visualized through a three-dimensional projection of the first three principal components, showing Clusters 0 and 1 as well-defined and distant (together 98.9% of the sample), while Cluster 2 occupied a peripheral position with atypical features (Figure 3).

### 4.4 Correlation analysis and characterization of clustered profiles

Correlation analysis revealed significant associations between metabolic and cardiovascular parameters. BMI showed a moderate positive correlation with blood glucose and triglycerides (*r* > 0.4, *p* < 0.01), indicating a shared pattern of metabolic alterations. Age correlated directly with systolic blood pressure (*r* = 0.38, *p* < 0.01), consistent with literature on cardiovascular risk progression in hypertensive adults.

**Figure 4:**
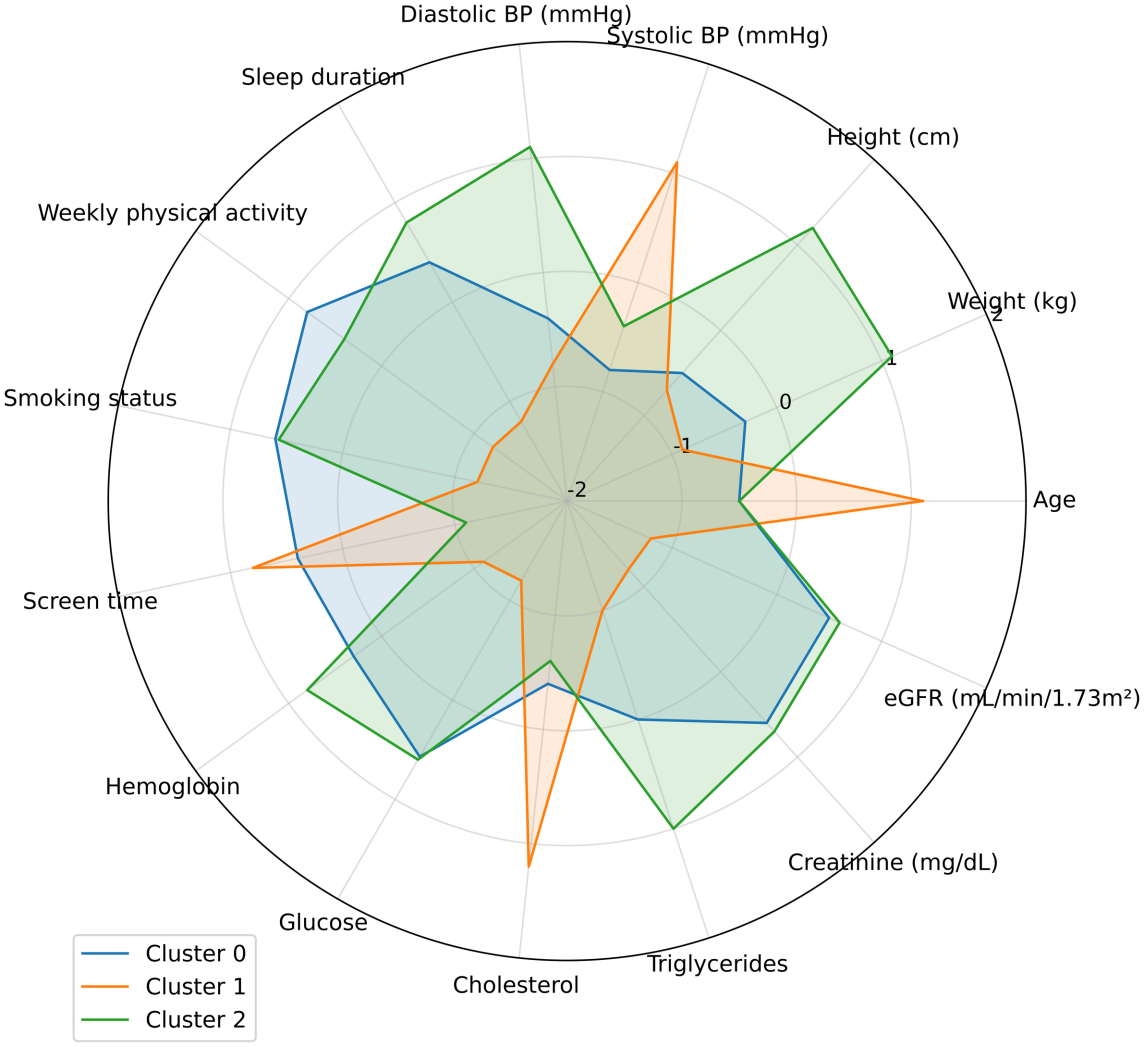
Heatmap of clinical and metabolic features by patient profile.

To facilitate interpretation of cluster-derived profiles, a multivariate heatmap summarized the main clinical, biochemical, and behavioral features of each group, illustrating variable magnitude differences across clusters. Key discriminators included blood glucose, cholesterol, hemoglobin, BMI, and physical activity (Figure 4).

Following cluster identification, each group was assigned a representative label based on its predominant pathophysiological and anthropometric traits, facilitating clinical interpretation and targeted intervention planning. Table 3 summarizes these phenotypes, integrating key metabolic, hematological, and morphological features into a concise overview.

**Table 3:** Nomenclature and key characteristics of patient profiles identified through clustering.

Group	Profile name	Main characteristics
0	Metabolic–hypertensive	Predominant glycemic alterations
1	Dyslipidemic–anemic	Elevated cholesterol and reduced hemoglobin
2	Constitutional–atypical	Distinctive anthropometric characteristics

### 4.5 Expert recommendation system

**Figure 5:**
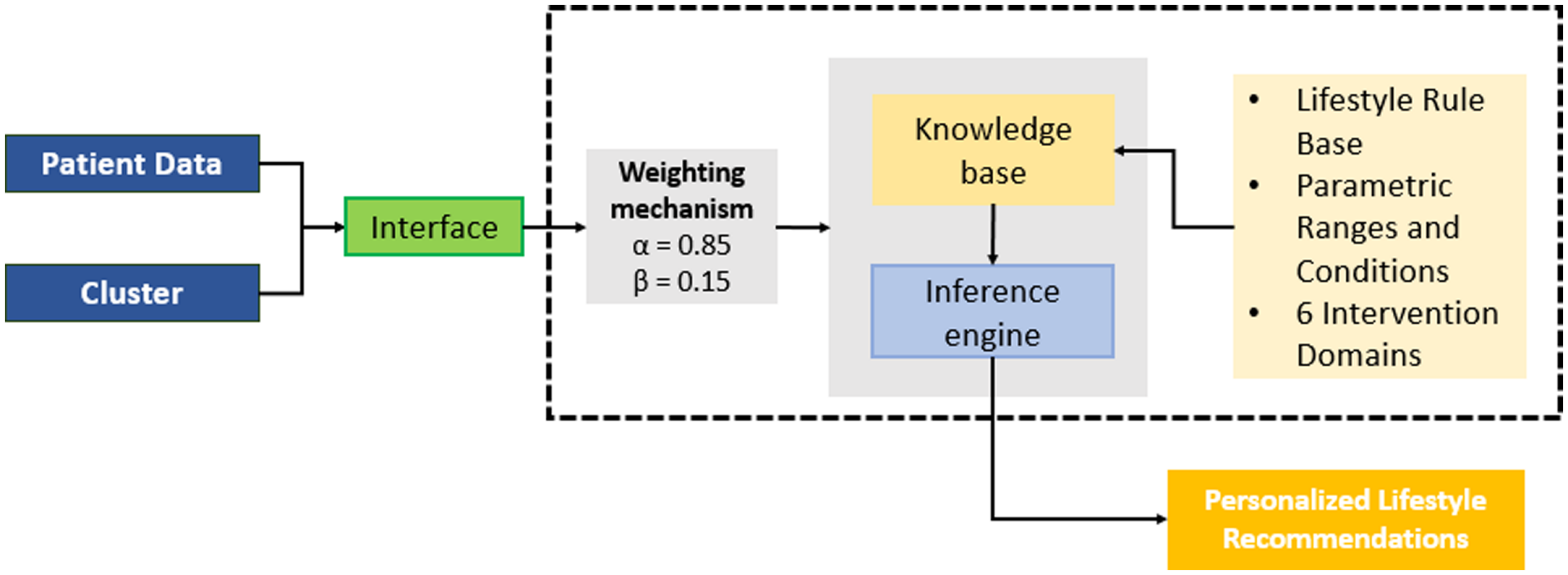
Modular architecture of the hybrid expert recommendation system integrating rule-based inference and clustering-derived patient profiles.

We implemented a hybrid architecture, combining a hierarchical rule-based inference engine with a clustering-derived pattern integration module. The knowledge base, structured in a six-dimension matrix (physical activity, stress management, diet, sleep patterns, therapeutic adherence, and general behaviors), applies specific rules to generate individualized recommendations based on clinical profile and cluster membership. Figure 5. Illustrates this modular structure.

Following system execution, a recommendation matrix classified outputs by intervention area and priority (Table 4). Physical activity: 51.7% increase, 45.6% maintain, 2.7% initiate. Stress management: 85.1% reduce, 14.1% maintain, 0.8% urgent. Diet: 62.0% restrict, 37.7% maintain, 0.3% increase. Sleep: 99.0% urgent, 1.0% general. Therapeutic adherence: 79.0% general, 9.1% maintain, 11.9% urgent. General behaviors: 78.3% urgent, 21.7% reduce. The prevalence of urgent recommendations in sleep, general behaviors, and stress management signals key intervention targets.

**Table 4:** Distribution of recommendations generated by area and intervention level.

Area	Recommendation	Percentage of patients (%)
Physical activity	Increase/improve	51.70
	Maintain/continue	45.60
	Initiate/implement	2.70
Stress management	Reduce/limit	85.10
	Maintain/continue	14.10
	Priority/urgent	0.80
Diet	Reduce/limit	62.00
	Maintain/continue	37.70
	Increase/improve	0.30
Sleep patterns	Priority/urgent	99.00
	General	1.00
Therapeutic adherence	General	79.00
	Maintain/continue	9.10
	Priority/urgent	11.90
General behaviors	Priority/urgent	78.30
	Reduce/limit	21.70

The predominance of urgent recommendations in sleep and general behavioral domains suggests that lifestyle-related risk factors remain substantially underaddressed in routine hypertension management. These findings highlight the need to prioritize structured behavioral assessment and targeted counseling within clinical workflows, particularly in settings where consultation time is limited and pharmacological treatment often dominates clinical attention.

**Figure 6:**
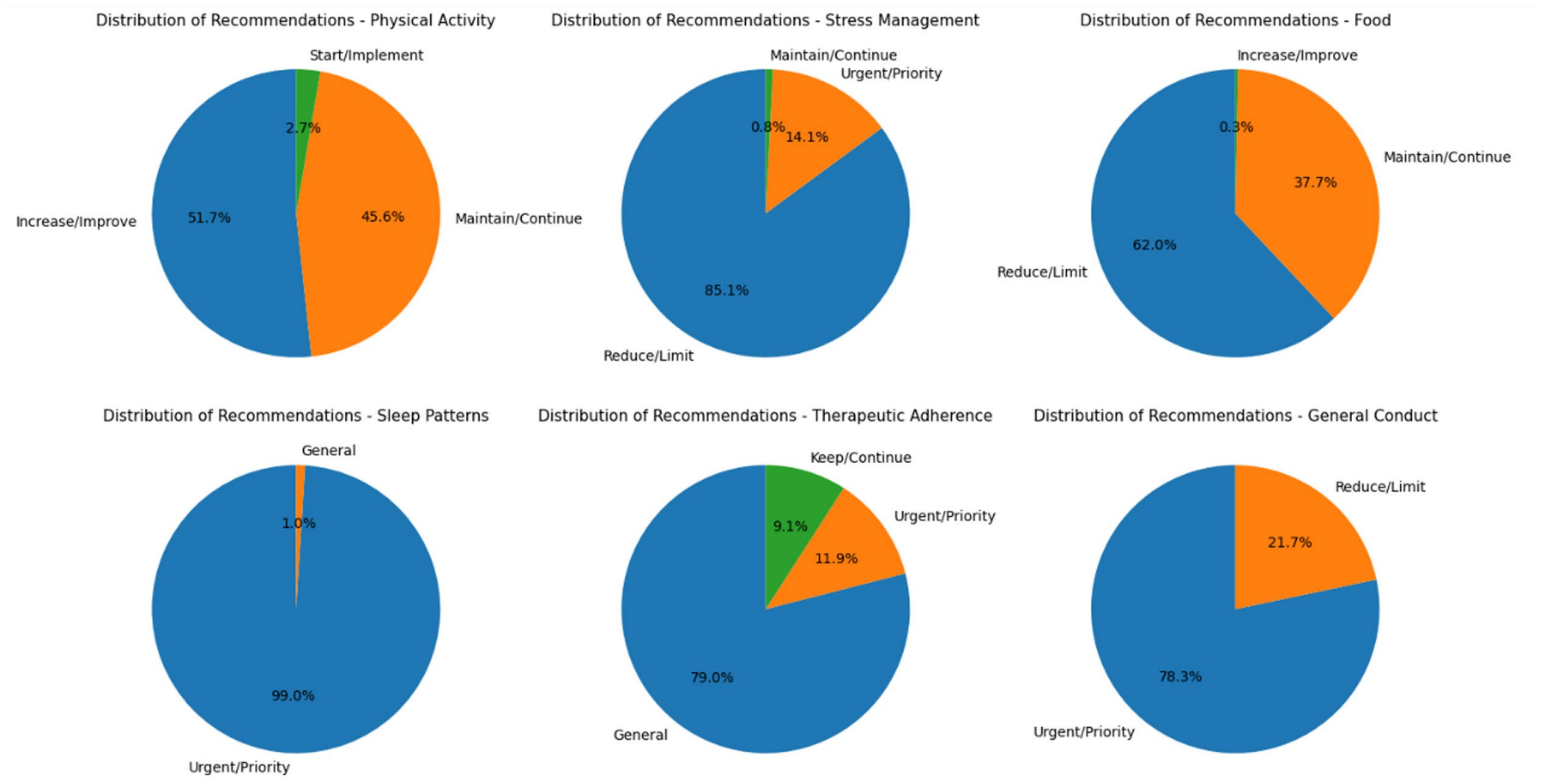
Percentage distribution of personalized recommendations by intervention category in hypertensive patients.

Sleep patterns showed a nearly universal need for urgent action (99.0%), underscoring an often-overlooked clinical factor. Therapeutic adherence was mostly general (79.0%), with 11.9% urgent. General behaviors presented 78.3% urgent cases, evidencing widespread harmful habits. Figure 6 illustrates the overall distribution by category and recommendation type.

The cluster-based recommendation analysis revealed substantial differences in the prioritized intervention areas for each group. As shown in Table 5, Cluster 0 predominantly received suggestions to increase physical activity (63.4%) and maintain current dietary habits (52.8%), reflecting metabolically active profiles with partially adequate habits yet with room for improvement. In contrast, Cluster 1—characterized by older age and dyslipidemia—concentrated recommendations on dietary reduction (85.2%) and urgent intervention in general behaviors (91.7%), highlighting elevated metabolic and behavioral risk. Cluster 2 exhibited an atypical pattern, with most recommendations oriented toward increasing physical activity (71.4%) and reducing harmful substance consumption (71.4%). Remarkably, this group received urgent adherence-to-treatment recommendations in 42.9% of cases, suggesting significant deficiencies in treatment compliance despite favorable anthropometric parameters.

**Table 5:** Distribution of recommendations by area and intervention level across clusters.

Category	Cluster 0	Cluster 1	Cluster 2
Physical activity	Increase (63.4%)	Maintain (58.3%)	Increase (71.4%)
Stress management	Reduce (82.7%)	Reduce (88.6%)	Maintain (57.1%)
Diet	Maintain (52.8%)	Reduce (85.2%)	Reduce (85.7%)
Sleep	Urgent (98.6%)	Urgent (99.6%)	Urgent (100%)
Therapeutic adherence	General (83.2%)	General (73.5%)	Urgent (42.9%)
General behaviors	Urgent (67.9%)	Urgent (91.7%)	Reduce (71.4%)

This multicriteria analysis reinforces the value of tailoring interventions to specific clinical and behavioral profiles. Table 5 summarizes the overall distribution of recommendations by area and intervention level across the three clusters.

To further illustrate the explainable interaction between clustering outputs and rule-based inference, representative patient cases were examined. For example, a typical Cluster 1 patient characterized by elevated cholesterol levels, reduced physical activity frequency, and suboptimal blood pressure control was assigned to the dyslipidemic–anemic profile. At the individual level, elevated lipid markers triggered dietary reduction rules, while reduced activity frequency activated physical activity maintenance or improvement rules. Cluster-level contextual weighting reinforced dietary prioritization due to the profile’s predominant metabolic characteristics. Consequently, the system generated a targeted recommendation emphasizing dietary restriction, stress reduction, and urgent sleep intervention.

These case-based illustrations demonstrate how individual indicators and cluster-derived patterns jointly contribute to interpretable recommendation generation. This comparative distribution underscores the distinct intervention priorities among the identified clusters. While certain domains—most notably sleep—consistently require urgent attention across all groups, other dimensions such as diet, physical activity, and general behaviors exhibit markedly differentiated patterns. These variations align closely with the underlying metabolic, cardiovascular, and behavioral profiles characterizing each cluster, reinforcing the need for targeted, profile-specific intervention strategies.

### 4.6 Expert system evaluation

The performance of the developed expert system was validated through a systematic comparison between its automated recommendations and the clinical judgment of specialists. A total of 120 cases were selected via stratified sampling based on the clustering profiles, ensuring proportional representation of the three groups previously identified in the cluster analysis. Each case was blindly assessed by five healthcare professionals—two cardiologists and three clinical nutritionists—each with over 5 years of experience in hypertension management. Clinical consensus was determined by simple majority; in cases of disagreement, the decision supported by at least two specialists was adopted, thereby enhancing objectivity and minimizing evaluation bias.

The analysis revealed an overall agreement rate of 78.3% between the expert system and the clinical consensus, with a Cohen’s Kappa coefficient of *κ* = 0.742 (95% CI: 0.678–0.806), representing a substantial agreement according to the Landis and Koch classification. These findings demonstrate the system’s high fidelity in replicating expert-level clinical decision-making. Table 6 presents a detailed breakdown of results by dimension, showing the distribution of recommendations generated by the system, the clinical consensus decisions, and the corresponding agreement percentages for each category.

**Table 6:** Detailed evaluation of agreement between expert system recommendations and clinical consensus.

Dimension	Recommendation	System *n* (%)	Consensus *n* (%)	Agreement (%)	κ
Physical activity	Initiate/implement	12 (10.0%)	15 (12.5%)	91.7	0.796
	Increase/improve	64 (53.3%)	59 (49.2%)	85.9	
	Maintain/continue	44 (36.7%)	46 (38.3%)	79.5	
Stress management	Priority/urgent	8 (6.7%)	12 (10.0%)	75.0	0.743
	Reduce/limit	94 (78.3%)	89 (74.2%)	83.0	
	Maintain/continue	18 (15.0%)	19 (15.8%)	84.2	
Nutrition	Increase/improve	5 (4.2%)	8 (6.7%)	62.5	0.681
	Maintain/continue	43 (35.8%)	47 (39.2%)	74.4	
	Reduce/limit	72 (60.0%)	65 (54.2%)	76.4	
Sleep patterns	General	8 (6.7%)	11 (9.2%)	72.7	0.784
	Priority/urgent	112 (93.3%)	109 (90.8%)	89.3	
Therapeutic adherence	Priority/urgent	16 (13.3%)	19 (15.8%)	68.4	0.698
	General	89 (74.2%)	86 (71.7%)	79.8	
	Maintain/continue	15 (12.5%)	15 (12.5%)	80.0	
General behaviors	Reduce/limit	31 (25.8%)	28 (23.3%)	71.0	0.634
	Priority/urgent	89 (74.2%)	92 (76.7%)	72.0	

The highest agreement was observed in sleep patterns (89.2%), followed by physical activity (83.3%) and stress management (81.7%). General behaviors showed the lowest concordance (71.7%), mainly due to differing thresholds for intervention in mixed alcohol–tobacco cases. The Cohen’s Kappa values ranged from 0.634 to 0.796, confirming robust system performance across all dimensions.

Cohen’s Kappa was used as the primary measure of agreement beyond chance between the expert system and clinical consensus (κ = 0.742). In addition, a Chi-square test was applied as a complementary analysis to assess statistical independence between recommendation distributions across intervention domains. We have not detect significant differences in any dimension, reinforcing the system’s validity in replicating expert judgment without systematic bias. Table 7 details the *χ*^2^ values and significance levels for each dimension.

**Table 7:** Chi-square test results by dimension.

Dimension	*χ* ^2^	gl	*p*-value
Physical activity	1.234	2	0.540
Stress management	2.891	2	0.236
Nutrition	3.672	2	0.159
Sleep patterns	0.445	1	0.505
Therapeutic adherence	1.789	2	0.409
General behaviors	0.287	1	0.592
Overall total	8.347	15	0.908

All *p*-values exceeded 0.05, supporting the null hypothesis of independence. This indicates that any discrepancies between the system’s recommendations and expert judgments are attributable to random variation rather than structural bias in the system’s decision logic.

The findings of this study—particularly the identification of three clinical-behavioral profiles through unsupervised learning and the subsequent generation of personalized recommendations—are aligned with the international trend toward adaptive digital health solutions. While studies such as Sookrah et al. (2019) have employed neural networks to personalize DASH diets with high accuracy, our work demonstrates that combining unsupervised clustering with a rule-based expert system can also produce valuable recommendations, even without large volumes of labeled training data. This hybrid approach facilitates personalized stratification in contexts where technical resources or data availability are limited, as is the case in many rural areas of Peru.

Compared to the digital intervention evaluated by Kario et al. (2021), which implemented a mobile chatbot with cloud-based monitoring, our system offers a more structured alternative that is less dependent on constant connectivity—an essential factor in technology-limited settings. The high agreement observed between the expert system and clinical judgment (*κ* = 0.742) supports its reliability, consistent with the findings of Islam and Shamsuddin (2021) in Bangladesh, where the use of CNNs enabled the identification of critical variables for lifestyle recommendation generation. However, unlike those studies, our proposal explicitly integrates behavioral factors and group profiles into the inferential logic, fostering a more holistic approach to managing hypertensive patients.

Despite global advances in the development of digital tools for hypertension management—such as the applications proposed by (Olivera Solís et al., 2022) in Cuba and (Alor-Hernandez et al., 2022) in Mexico—scientific output in the Peruvian context remains scarce, particularly regarding approaches that simultaneously address patient segmentation and automated recommendation generation. The present proposal addresses this gap by integrating clustering algorithms, expert system architecture, and robust empirical evaluation. In doing so, it represents a relevant and replicable contribution for primary healthcare systems in Latin America, especially for the non-pharmacological management of chronic diseases such as hypertension.

## 5 Discussion

This section interprets the methodological and clinical implications of the findings, contrasts the proposed approach with existing AI-based hypertension management systems, and critically examines limitations and future research directions.

The present study demonstrates that integrating unsupervised patient stratification with a rule-based expert inference system constitutes a viable and effective approach for generating personalized lifestyle recommendations in hypertensive patients. Unlike conventional digital health solutions that rely primarily on supervised prediction or user-centric mobile applications, the proposed hybrid architecture emphasizes decision-support logic, interpretability, and scalability under data and resource constraints.

### 5.1 Value of unsupervised stratification for clinical decision support

The use of unsupervised learning enabled the identification of three clinically meaningful patient profiles without requiring predefined labels or outcome annotations. This finding is consistent with prior evidence suggesting that latent structures in heterogeneous clinical datasets can be exploited to support patient stratification and personalized intervention design (Karaçam et al., 2025; Sonnweber et al., 2023).

In contrast to supervised hypertension prediction models—often focused on binary risk classification—the clustering-based approach adopted in this study captures multidimensional clinical–behavioral patterns, allowing recommendations to be contextualized beyond isolated risk factors. This is particularly relevant in real-world healthcare environments, where incomplete labeling, variable measurement protocols, and heterogeneous populations limit the applicability of purely supervised pipelines.

### 5.2 Hybrid AI as an alternative to black-box recommendation systems

A central contribution of this work lies in the hybrid integration of machine learning and explicit clinical reasoning. While recent studies have reported high accuracy using deep learning models for hypertension-related tasks, such approaches frequently operate as black boxes, limiting clinical interpretability and trust (Agarwal et al., 2026; Leiherer et al., 2026).

Unlike deep learning architectures that prioritize predictive accuracy, our hybrid model emphasizes transparent rule-based reasoning supported by unsupervised stratification. Similarly, in contrast to application-centric interventions such as mobile apps or chatbots, which depend on sustained user engagement and connectivity, the proposed system focuses on backend decision intelligence integrated into clinical workflows. This methodological distinction positions the system as a complementary alternative to predictive and engagement-driven paradigms, particularly in healthcare environments with limited technological infrastructure.

By contrast, the proposed system decouples pattern discovery (via PCA and clustering) from decision logic (via rule-based inference), enabling transparent mapping between patient characteristics and generated recommendations. This design aligns with emerging informatics frameworks that advocate for explainable and auditable decision-support systems, particularly in clinical contexts where accountability and traceability are essential.

To assess the added value of unsupervised stratification, a baseline configuration relying solely on rule-based inference (*α* = 1.0; *β* = 0.0) was explored during model development. While this approach produced technically valid recommendations, it resulted in more fragmented decision patterns in heterogeneous patient cases, lacking contextual coherence across clinically similar profiles. Incorporating cluster-derived weighting improved consistency within phenotypically related subgroups, supporting the integrative contribution of the hybrid architecture beyond standalone rule-based reasoning.

From a clinical adoption perspective, interpretability plays a decisive role in physician trust and workflow integration. Rule-based reasoning allows healthcare professionals to trace each recommendation back to explicit clinical indicators and predefined thresholds, facilitating transparency and accountability. In contrast, black-box deep learning models may achieve high predictive performance but often lack explanatory clarity, which can hinder acceptance in routine clinical environments. By maintaining explicit logical pathways, the proposed hybrid architecture aligns more closely with clinician-centered decision-making processes.

### 5.3 Comparison with application-centric and chatbot-based interventions

Many digital interventions for hypertension management have been implemented as mobile applications, chatbots, or IoT-enabled platforms, focusing on user engagement and continuous self-monitoring. Although these systems have demonstrated positive behavioral outcomes, they often depend on sustained connectivity, frequent user interaction, and technological infrastructure (Colakoglu et al., 2025; Peerbolte et al., 2025).

The approach proposed in this study differs in that it prioritizes backend decision intelligence over frontend interaction. Recommendations are generated based on structured clinical data and expert knowledge, without requiring constant user input or real-time connectivity. This characteristic enhances its suitability for resource-limited or rural healthcare settings, where digital access may be intermittent and healthcare staff workloads are high.

### 5.4 Clinical alignment and expert validation as a strength of the model

The validation results indicate substantial agreement between the expert system’s recommendations and clinical expert consensus (*κ* = 0.742), supporting the system’s reliability as a decision-support tool rather than a diagnostic substitute. Comparable agreement levels have been reported in studies validating clinical decision-support systems against expert judgment, reinforcing the appropriateness of expert-based validation frameworks (Bouattour et al., 2026; Brand et al., 2025).

This level of agreement represents a methodological strength of the study. Unlike many AI-based health systems that report predictive accuracy against labeled datasets, our evaluation framework directly measures concordance with multidisciplinary clinical judgment. By anchoring system performance to expert consensus rather than purely statistical metrics, we reinforce the system’s practical relevance, interpretability, and clinical credibility.

Notably, the highest concordance was observed in domains such as sleep patterns and physical activity, highlighting the system’s ability to capture lifestyle dimensions that are often underemphasized in traditional hypertension management. Lower agreement in general behaviors reflects known subjectivity in assessing mixed risk behaviors, an issue also reported in previous behavioral health informatics studies (Abinaya and Rajesh, 2026).

### 5.5 Implications for non-pharmacological hypertension management

From a public health and informatics perspective, the proposed system addresses a critical gap in non-pharmacological hypertension management by offering scalable, personalized, and explainable recommendations without increasing clinician workload. The high prevalence of urgent recommendations related to sleep, stress, and general behaviors underscores the importance of incorporating lifestyle-focused decision support alongside pharmacological treatment (Akik et al., 2024).

Moreover, the system’s architecture allows for incremental refinement of the rule base and adaptation to different clinical contexts without retraining machine learning models, an advantage over purely data-driven systems when deployed in evolving healthcare environments.

### 5.6 Limitations and future research directions

Despite its strengths, this study has limitations that should be acknowledged. The dataset was derived from a single regional healthcare context (San Martín, Peru), which may limit direct generalizability of the identified phenotypes and calibrated rule thresholds to other populations. However, the proposed architecture is modular and transportable: the unsupervised stratification layer can be recalibrated using locally available data, and the rule-based inference engine can be adapted according to context-specific clinical guidelines. This design supports methodological transferability even if phenotype prevalence and healthcare infrastructure differ across settings.

Additionally, the rule base was constructed through collaboration with specialists operating within the same regional healthcare environment, which may introduce contextual bias in threshold calibration and intervention prioritization. Although the rules were grounded in international guidelines, their operational interpretation reflects local clinical practice patterns. Consequently, external validity may be influenced by regional epidemiological characteristics and healthcare infrastructure differences. Replication in diverse geographic and healthcare settings is necessary to evaluate cross-context generalizability.

Future research should explore the integration of temporal data to capture longitudinal patient trajectories, as well as the incorporation of explainability techniques to further enhance transparency. Comparative studies evaluating hybrid systems against fully supervised or deep learning-based approaches under equivalent conditions would also provide valuable insights (Chen et al., 2009; Kadum et al., 2023; Montagna et al., 2023).

## 6 Conclusion

This section summarizes the main contributions of the study and outlines potential avenues for clinical implementation and future methodological refinement.

This study demonstrates that integrating unsupervised patient stratification with rule-based clinical reasoning enables the development of an interpretable and scalable decision-support system for personalized lifestyle recommendations in hypertensive patients. The hybrid architecture balances data-driven profiling with explicit expert logic, achieving substantial agreement with multidisciplinary clinical consensus (*κ* = 0.742) and reinforcing its clinical credibility.

From a public-health perspective, the proposed system provides a structured and scalable framework for strengthening non-pharmacological hypertension management in resource-limited settings. By enabling standardized lifestyle assessment and recommendation generation without requiring large labeled datasets or continuous digital engagement, the model promotes more equitable and consistent clinical decision support in regions with limited specialist availability.

The architecture is adaptable beyond the regional context in which it was developed. Because the system decouples unsupervised patient stratification from rule-based inference, it can be recalibrated using locally available clinical data and guideline-based thresholds in diverse healthcare environments. Moreover, the hybrid framework may be extended to support non-pharmacological management of other chronic conditions—such as diabetes, obesity, or broader cardiovascular risk syndromes—by redefining domain-specific variables and expert rules while preserving the core stratification–inference structure.

Future developments may include integration with electronic health record (EHR) systems to enable automated data ingestion and real-time recommendation updates. Incorporating longitudinal patient trajectories would support dynamic profile evolution and continuous lifestyle adjustment, aligning the framework with emerging digital health and learning health system paradigms.

## Data Availability

The raw data supporting the conclusions of this article will be made available by the authors, without undue reservation.

## References

[ref1] Abinaya G. Rajesh K. (2026). Machine learning algorithm benchmarking for cardiovascular risk prediction, In: Rathore, V.S., Piuri, V., Babo, R., and Karthik, S. (eds) Universal Threats in Expert Applications and Solutions (UNI-TEAS 2025). Lecture Notes in Networks and Systems. Singapore: Springer. 1452, 163–179. doi: 10.1007/978-981-96-7292-9_14

[ref2] Agarwal K. Mahalle P. N. Thorat B. D. Mane V. (2026). Heart failure prediction with explainable artificial intelligence towards trusted approach: a comparative analysis of black-box and transparent models, In: Tuba, M., Akashe, S., and Joshi, A. (eds) ICT Systems and Sustainability (ICT4SD 2025). Lecture Notes in Networks and Systems. Cham: Springer. 1649, 262–274. doi: 10.1007/978-3-032-06674-9_27

[ref3] Akik C. El-Dirani Z. Willis R. Truppa C. Zmeter C. Aebischer-Perone S. . (2024). Providing continuity of care for people living with noncommunicable diseases in humanitarian settings: a qualitative study of health actors’ experiences in Lebanon. J. Migr. Health 10. doi: 10.1016/j.jmh.2024.100269 PMC11915524 40103922

[ref4] Alor-Hernandez J. A. Cruz Ramos N. A. Alor-Hernández G. Sánchez-Cervantes J. L. Rodríguez-Mazahua L. (2022). Desarrollo de Un Módulo Para La Prevención de La Hipertensión Usando El Paradigma IoT y Aprendizaje Automático. Res. Comput. Sci. 151, 91–104.

[ref5] Andersson U. Nilsson P. M. Kjellgren K. Hoffmann M. Wennersten A. Midlöv P. (2023). PERson-centredness in hypertension management using information technology: a randomized controlled trial in primary care. J. Hypertens. 41, 246–253. doi: 10.1097/HJH.0000000000003322, 36394295 PMC9799039

[ref6] Athanasakis K. (2017). The socioeconomic effects of uncontrolled hypertension. Curr. Vasc. Pharmacol. 16, 5–9. doi: 10.2174/1570161115666170413145125, 28412912

[ref7] Boehme A. K. Esenwa C. Elkind M. S. V. (2017). Stroke risk factors, genetics, and prevention. Circ. Res. 120, 472–495. doi: 10.1161/CIRCRESAHA.116.308398, 28154098 PMC5321635

[ref8] Bouattour Y. Maâloul M. H. Bahloul Z. Marzouk S. (2026). A machine learning–based method for supporting the diagnosis of eosinophilic granulomatosis with polyangiitis: development and evaluation. Intell.-based Med. 13:100317. doi: 10.1016/j.ibmed.2025.100317

[ref9] Brand L. Mitrov-Winkelmolen L. Kuijper T. M. Bosch T. M. Krens L. L. (2025). Evaluation and validation of a clinical decision support system for dose optimisation in hospitalized patients with (morbid) obesity – a retrospective, observational study. BMC Med. Inform. Decis. Mak. 25:140. doi: 10.1186/s12911-025-02963-3, 40108614 PMC11921672

[ref10] Cajita M. I. Zheng Y. Kariuki J. K. Vuckovic K. M. Burke L. E. (2021). MHealth technology and CVD risk reduction. Curr. Atheroscler. Rep. 23:36. doi: 10.1007/s11883-021-00927-2, 33983491

[ref11] Caliñski T. Harabasz J. (1974). A dendrite method foe cluster analysis. Commun. Stat. 3, 1–27. doi: 10.1080/03610927408827101

[ref12] Carey R. M. Muntner P. Bosworth H. B. Whelton P. K. (2018). Prevention and control of hypertension. J. Am. Coll. Cardiol. 72, 1278–1293. doi: 10.1016/j.jacc.2018.07.008, 30190007 PMC6481176

[ref13] Chen Y.-F. Jiang X. Huang Y.-F. Hsu Y.-N. Lin H.-H. (2009). Design of clinical support systems using integrated genetic algorithm and support vector machine. Lect. Notes Comput. Sci. Incl. Subser. Lect. Notes Artif. Intell. Lect. Notes Bioinform. 5702, 935–944. doi: 10.1007/978-3-642-03767-2_96

[ref14] Choudhry N. K. Kronish I. M. Vongpatanasin W. Ferdinand K. C. Pavlik V. N. Egan B. M. . (2022). Medication adherence and blood pressure control: a scientific statement from the American Heart Association. Hypertension 79, e1–e14. doi: 10.1161/HYP.0000000000000203, 34615363 PMC11485247

[ref15] Colakoglu S. Durmus M. Polat Z. P. Yildiz A. Sezgin E. (2025). User engagement with a multimodal conversational agent for self-care and chronic disease management: a retrospective analysis. J. Med. Syst. 49:76. doi: 10.1007/s10916-025-02202-2, 40488988 PMC12148993

[ref16] Davies D. L. Bouldin D. W. (1979). A cluster separation measure. IEEE Trans. Pattern Anal. Mach. Intell. PAMI-1, 224–227. doi: 10.1109/TPAMI.1979.4766909, 21868852

[ref17] Gheorghe A. Griffiths U. Murphy A. Legido-Quigley H. Lamptey P. Perel P. (2018). The economic burden of cardiovascular disease and hypertension in low- and middle-income countries: a systematic review. BMC Public Health 18:975. doi: 10.1186/s12889-018-5806-x, 30081871 PMC6090747

[ref18] Goorani S. Zangene S. Imig J. D. (2024). Hypertension: a continuing public healthcare issue. Int. J. Mol. Sci. 26:123. doi: 10.3390/ijms26010123, 39795981 PMC11720251

[ref19] Hajat C. Stein E. (2018). The global burden of multiple chronic conditions: a narrative review. Prev. Med. Rep. 12, 284–293. doi: 10.1016/j.pmedr.2018.10.008, 30406006 PMC6214883

[ref20] Hernández-Vásquez A. Carrillo Morote B. N. Azurin Gonzales V. d. C. Turpo Cayo E. Y. Azañedo D. (2023). Análisis Espacial de La Hipertensión Arterial En Adultos Peruanos, 2022. Arch. Peru. Cardiol. Cir. Cardiovasc. 4, 48–54. doi: 10.47487/apcyccv.v4i2.296, 37780947 PMC10538923

[ref21] Islam M. M. Shamsuddin R. (2021). Machine learning to promote health management through lifestyle changes for hypertension patients. Array 12:100090. doi: 10.1016/j.array.2021.100090

[ref22] Kadum S. Y. Salman O. H. Taha Z. K. Said A. B. Ali M. A. M. Qassim Q. S. . (2023). Machine learning-based telemedicine framework to prioritize remote patients with multi-chronic diseases for emergency healthcare services. Netw. Model. Anal. Health Inform. Bioinform. 12. doi: 10.1007/s13721-022-00407-w

[ref23] Karaçam M. Kültürsay B. Mutlu D. Tanyeri S. Kaya A. Efe S. Ç. . (2025). From patterns to prognosis: machine learning–derived clusters in advanced heart failure. Front. Cardiovasc. Med. 12. doi: 10.3389/fcvm.2025.1669538, 41210338 PMC12589050

[ref24] Kario K. Nomura A. Harada N. Okura A. Nakagawa K. Tanigawa T. . (2021). Efficacy of a digital therapeutics system in the Management of Essential Hypertension: the HERB-DH1 pivotal trial. Eur. Heart J. 42, 4111–4122. doi: 10.1093/eurheartj/ehab559, 34455443 PMC8530534

[ref25] Leiherer A. Schnetzer L. Mink S. Mader A. Mündlein A. Bermeitinger B. . (2026). Interpretable type 2 diabetes incidence prediction with AutoScore: a model based on standard clinical parameters. Int. J. Med. Inform. 206:106161. doi: 10.1016/j.ijmedinf.2025.106161, 41176846

[ref26] Lozano-Flores E. D. M. (2023). Application of artificial intelligence techniques in studies on eating habits: bibliometric analysis. Revista Científica de Sistemas e Informática 3:e489. doi: 10.51252/rcsi.v3i1.489

[ref27] Mash R. Schouw D. Fischer A. E. (2022). Evaluating the implementation of the GREAT4Diabetes WhatsApp Chatbot to educate people with type 2 diabetes during the COVID-19 pandemic: convergent mixed methods study. JMIR Diabetes 7:e37882. doi: 10.2196/37882, 35537057 PMC9236126

[ref28] Montagna S. Pengo M. F. Ferretti S. Borghi C. Ferri C. Grassi G. . (2023). Machine learning in hypertension detection: a study on world hypertension day data. J. Med. Syst. 47:1. doi: 10.1007/s10916-022-01900-5, 36580140 PMC9800348

[ref29] Olivera Solís R. A. Reyes Morel D. García Ocaña J. A. González Rodríguez E. Garí Llanes M. (2022). Aplicación Innovadora Para Dispositivos Android Para El Diagnóstico, Evaluación y Tratamiento de La Hipertensión Arterial. Rev. Ing. Electrón. Autom. Comun. 43, 1–15.

[ref30] Peacock E. Craig L. S. Krousel-Wood M. (2022). Electronic health strategies to improve medication adherence in patients with cardiometabolic disease: current status and future directions. Curr. Opin. Cardiol. 37, 307–316. doi: 10.1097/HCO.0000000000000971, 35731675 PMC9228772

[ref31] Peerbolte T. F. van Diggelen R. J. A. van den Haak P. Geurts K. Evers L. J. W. Bloem B. R. . (2025). Conversational agents supporting self-Management in People with a chronic disease: systematic review. J. Med. Internet Res. 27:e72309. doi: 10.2196/72309, 40857094 PMC12421203

[ref32] Rousseeuw P. J. (1987). Silhouettes: a graphical aid to the interpretation and validation of cluster analysis. J. Comput. Appl. Math. 20, 53–65. doi: 10.1016/0377-0427(87)90125-7

[ref33] Ruiz-Alejos A. Carrillo-Larco R. M. Bernabé-Ortiz A. (2022). Prevalencia e Incidencia de Hipertensión Arterial En Perú: Revisión Sistemática y Metaanálisis. Rev. Peru Med. Exp. Salud Publica 38, 521–529. doi: 10.17843/rpmesp.2021.384.8502, 35385004

[ref34] Saucedo G. Friso F. Politi M. (2021). Implementación y Funcionamiento de Un Sistema de Información Clínica En Una Comunidad Terapéutica. Rev. Cient. Sist. Inform. 1, 37–50. doi: 10.51252/rcsi.v1i1.109

[ref35] Schoenthaler A. Knafl G. J. Fiscella K. Ogedegbe G. (2017). Addressing the social needs of hypertensive patients. Circ. Cardiovasc. Qual. Outcomes 10, e003659. doi: 10.1161/CIRCOUTCOMES.117.003659, 28830861 PMC5571828

[ref36] Sonnweber T. Tymoszuk P. Steringer-Mascherbauer R. Sigmund E. Porod-Schneiderbauer S. Kohlbacher L. . (2023). The combination of supervised and unsupervised learning based risk stratification and phenotyping in pulmonary arterial hypertension—a long-term retrospective multicenter trial. BMC Pulm. Med. 23:143. doi: 10.1186/s12890-023-02427-2, 37098543 PMC10131314

[ref37] Sookrah R. Dhowtal J. D. Nagowah S. D. (2019). “A DASH diet recommendation system for hypertensive patients using machine learning,” in 2019 7th International Conference on Information and Communication Technology (ICoICT), (New York, NY, USA: IEEE), 1–6.

[ref38] Yan Y. Chen R. Yang Z. Ma Y. Huang J. Luo L. . (2022). Application of Back propagation neural network model optimized by particle swarm algorithm in predicting the risk of hypertension. J. Clin. Hypertens. 24, 1606–1617. doi: 10.1111/jch.14597, 36380516 PMC9731601

