# Supplementary figures and images for: Hybrid expert system for lifestyle recommendations in hypertensive patients
Source: Front Artif Intell. 2026 Mar 30;9:1794925. doi: 10.3389/frai.2026.1794925 (PMC13074202; doi:10.3389/frai.2026.1794925)

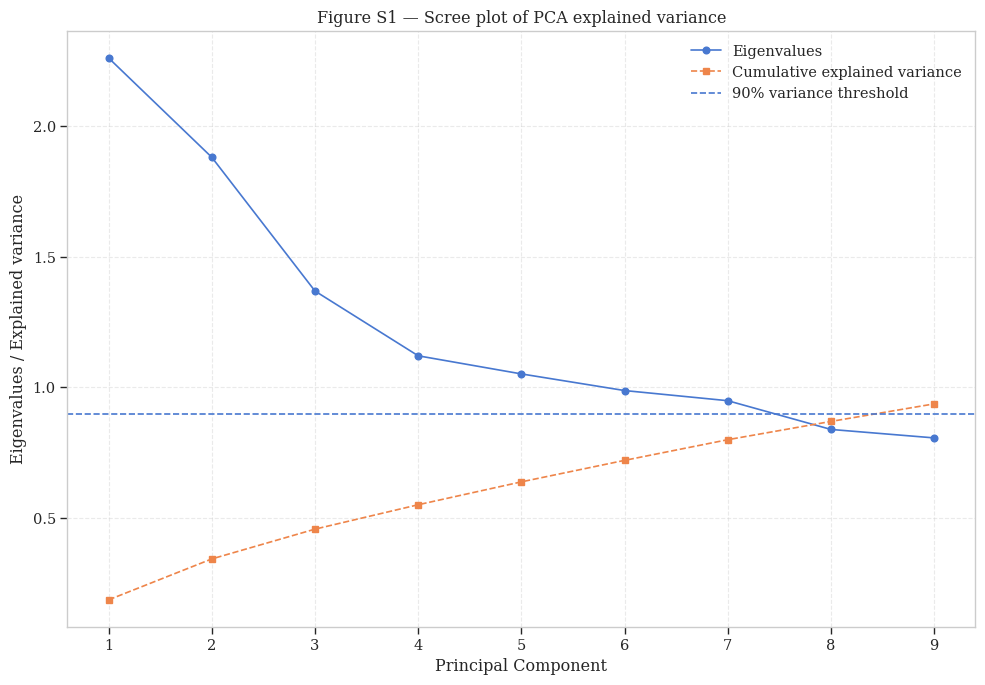

Supplement: Supplementary file 1 [file Image_1.PNG]

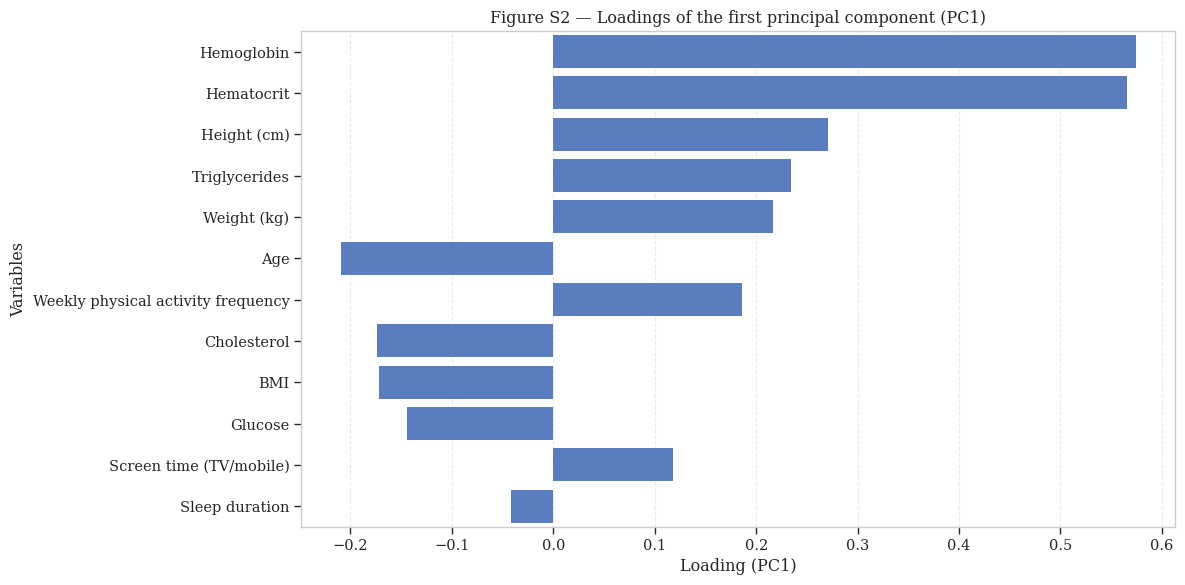

Supplement: Supplementary file 2 [file Image_2.PNG]

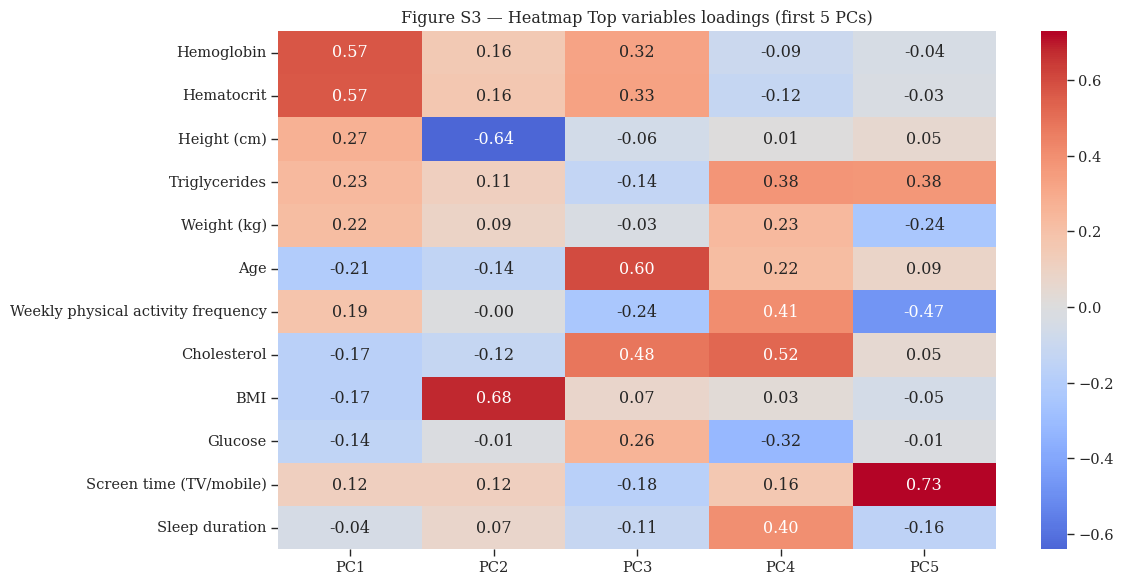

Supplement: Supplementary file 3 [file Image_3.PNG]
